# Impact of depressive symptoms on adverse effects in people with epilepsy on antiseizure medication therapy

**DOI:** 10.1002/epi4.12943

**Published:** 2024-04-16

**Authors:** Jürgen Panholzer, Amadeus Hauser, Nadia Thamm, Gudrun Gröppel, Kurosch Yazdi‐Zorn, Tim J. von Oertzen

**Affiliations:** ^1^ Department of Neurology Kepler University Hospital Linz Austria; ^2^ Faculty of Medicine Johannes Kepler University Linz Austria; ^3^ Department of Pediatrics and Adolescent Medicine Kepler University Hospital Linz Austria; ^4^ Department of Psychiatry – Specialization Addiction Medicine Kepler University Hospital Linz Austria

**Keywords:** adverse effects, antiseizure medication, depression, epilepsy, human

## Abstract

**Objective:**

We studied the impact of depressive symptoms on adverse effects (AEs) in people with epilepsy (PWE) on antiseizure medication (ASM) therapy. An effect of depression on the AE burden has already been reported. We studied the correlation of various depressive symptoms with specific AEs to assess which AEs are especially prone to being confounded by particular depressive symptoms.

**Methods:**

PWE filled in a variety of questionnaires including the “Neurological Disorder Depression Inventory for Epilepsy” (NDDI‐E), “Emotional Thermometers 4” (ET4) and “Liverpool Adverse Events Profile” (LAEP). Depression was defined by a NDDI‐E score > 13. Depressive symptoms consisted of NDDI‐E and ET4 items. Discriminant analysis identified those AEs (=LAEP items) that were most highly influenced by depression. Logistic regression analysis yielded correlations of different depressive symptoms with specific AEs.

**Results:**

We included 432 PWE. The strongest discriminators for depression were the LAEP items “Depression”, “Nervousness/agitation,” and “Tiredness”. Out of all depressive symptoms “Everything I do is a struggle” most strongly correlated with total LAEP score (odds ratio [OR] = 3.1) and correlated with all but one LAEP item. Other depressive symptoms correlated to varying degrees with total LAEP and item scores. The number of ASMs, lack of seizure remission, and female gender correlated with high LAEP scores.

**Significance:**

To the best of our knowledge, we are the first to show that various depressive symptoms correlate with specific LAEP items. This information can be helpful for quick evaluation of whether the reporting of different LAEP items may be confounded by particular depressive symptoms. This is relevant because changes in therapy may differ depending on if AEs are confounded by depressive symptoms. Simply reporting a particular depressive symptom may give a clue to whether specific AEs are confounded by depression. Our findings confirm the importance of screening for depression in all PWE.

**Plain Language Summary:**

In this study we measured depressive disorder and side effects caused by medication used to treat epilepsy with self‐reported questionnaires in a cohort of people with epilepsy. We found depressive disorder to influence the perception of side effects that are caused by drugs used to treat epilepsy. This knowledge can help to identify if the reporting of side effects is influenced by depression. Treating depression may help to reduce side effects and may thus increase the tolerability of anti‐epileptic medication. People who tolerate their medication are more likely to take it and are thus less likely to develop epileptic seizure.

**Key Points**

- Different depressive symptoms correlate with different LAEP item scores
- NDDI‐E item “Everything I do is a struggle” correlates most strongly with total LAEP and most item scores
- Reporting of particular depressive symptoms can give a clue to whether specific AEs are confounded
- We confirm the importance of screening for depression in all PWE

## 1 INTRODUCTION

Antiseizure medications (ASM) are linked with different adverse effects (AEs) including those related with emotion and physical symptoms. Therefore each drug should be carefully selected depending on patients' individual needs.^1^ Intolerable AEs may lead to the discontinuation of drug therapy and therefore play an important role in preventing seizure remission.^2^ Some of the more recently developed ASM causes less AE burden and can significantly increase therapy adherence, leading to better outcomes.^3^ Specific methods for assessing AEs such as the Liverpool Adverse Event Profile (LAEP) help to recognize and reduce AEs.^4^

Patients with epilepsy (PWE) have an up to 4‐fold higher risk of developing psychiatric disorders such as depression than the healthy population.^5^ This is important as previous studies report a correlation of depression with high total LAEP scores.^6^, ^7^ Kim et al. report the prediction of LAEP items related to emotion and cognition by depression.^8^ While the cause‐and‐effect relationship is uncertain, a confounding effect of depression on LAEP scores is likely. Thus, increased perception of AEs due to depression may affect therapy adherence and increase the burden of epilepsy.

Our study attempts to further analyze the relationship between depression and AEs in PWE on ASM therapy, building on the already confirmed impact of depression on total LAEP scores. We assess the correlation of particular depressive symptoms with LAEP item scores. The aim of our study is to elaborate which LAEP items are especially prone to being confounded by particular depressive symptoms. This is relevant because optimal therapy may differ depending on if AEs are confounded by depressive symptoms or are solely caused by ASM.

## 2 MATERIALS AND METHODS

This cross‐sectional study was approved by the ethic committee of the medical faculty of the Johannes Kepler University Linz. All participating patients signed informed consent forms and accurately completed the questionnaires.

### 2.1 Patient sample

During a one‐year period all patients attending the epilepsy outpatient clinic of the department of Neurology 1, Kepler University Hospital, were asked to fill in three different questionnaires, the “Liverpool Adverse Events Profile” (LAEP),^4^ “Neurological Disorder Depression Inventory for Epilepsy” (NDDI‐E),^6^ and “Emotional Thermometer 4” (ET4).^9^ Demographic and clinical variables were extracted from digital patient records. Inclusion criteria were age equal or greater than 18 years, diagnosis of epilepsy and long‐term ASM therapy. Patients who did not complete the questionnaires were excluded. If more than one visit occurred during the study period, only the questionnaires of the first visit were considered.

### 2.2 Study design

Initially, we assessed the correlation of total AE burden (=total LAEP score) with demographic and clinical variables. Depression was defined by a total NDDI‐E score > 13. Depressive symptoms were made up of NDDI‐E and ET4 items. We identified the AEs that were most susceptible to be influenced by depression using discriminant analysis.

In a last step, we studied the correlation of various depressive symptoms (=NDDI‐E and ET4 item scores) with specific AEs (=LAEP item scores) via logistic regression analysis.

### 2.3 Questionnaires

#### 2.3.1 Adverse effects

The LAEP is a well‐established self‐reported questionnaire used for detecting AEs in PWE on ASM therapy.^10^ It consists of 19 different items, each representing an AE typical for ASM therapy. These items are assessed by a four item Likert scale (1 = “never” a problem, 2 = “rarely”, 3 = “sometimes”, and 4 = “always”). The scores of all items can be added up (=total AE burden) but each single item (=single AEs) can be analyzed too. Total AE burden over 45 is regarded as high.^4^

#### 2.3.2 Depression

the NDDI‐E is a robust self‐reported tool for detecting major depressive disorder in PWE.^6^ Six items are assessed by a four item Likert‐scale. All items represent typical symptoms of major depressive disorder. An overall score of >15 enables the detection of major depressive episodes with more than 90% specificity, 81% sensitivity and a positive predictive value of 62%. Validation of the German version showed that the best cut off regarding the balance of median sensitivity and specificity is at >13.^11^, ^12^ As we used the German version, we used the cutoff at >13. The ET4 is a screening tool consisting of visual analogue scales for detection of depression, anxiety, distress, and aggression. Originally, it was developed for use in neuro‐oncology but was later validated in epilepsy as well.^9^ In our study we use the ET items “anxiety” and “anger” as potential symptoms of depression. A cut‐off of >5 marks a significant magnitude of either “anxiety” or “anger” with 10 being the maximal value.

### 2.4 Statistical analyses

#### 2.4.1 Univariate analysis of demographic and clinical variables

Firstly, we studied the correlation of total AE burden with demographic and clinical variables including parameters for epilepsy and depression. Group 1 (low burden) was defined as having an LAEP score ≤ 44 and group 2 as an LAEP score > 44 (high burden). According to the type and distribution of variables either Chi‐square analysis, Student's *t*‐test or Mann–Whitney *U*‐test was used.

#### 2.4.2 Discriminant analysis of LAEP items and depression

Discriminant analysis was used to identify those AEs that are most highly influenced by depression. While this method was not able to show causal relationship, it gave insight into the potential confounding effect of depression on reporting of specific AEs. Higher correlation coefficients are suggestive of a higher likelihood of depression being present.

#### 2.4.3 Logistic regression of LAEP items and depressive symptoms

In the last step, we studied the correlation of NDDI‐E and ET items with LAEP items. This gives insight into the potential confounding effect of particular depressive symptoms on specific AEs. For each AE two groups and separate logistic regression models were created. Group 1 (low burden) was defined by a score of 1–3 and group 2 (high burden) by a score of 4. The individual AEs were the dependent variables (group 1, group 2), while NDDI‐E and ET items were the independent variables. Further independent variables were age, gender, epilepsy type (generalized, focal, unknown), seizure remission for at least 12 months (yes, no) and number of ASM. Statistical analysis was done using the Statistical Package for Social Sciences (SPSS, version 25, windows 10).

## 3 RESULTS

### 3.1 Patient inclusion

**FIGURE 1:**
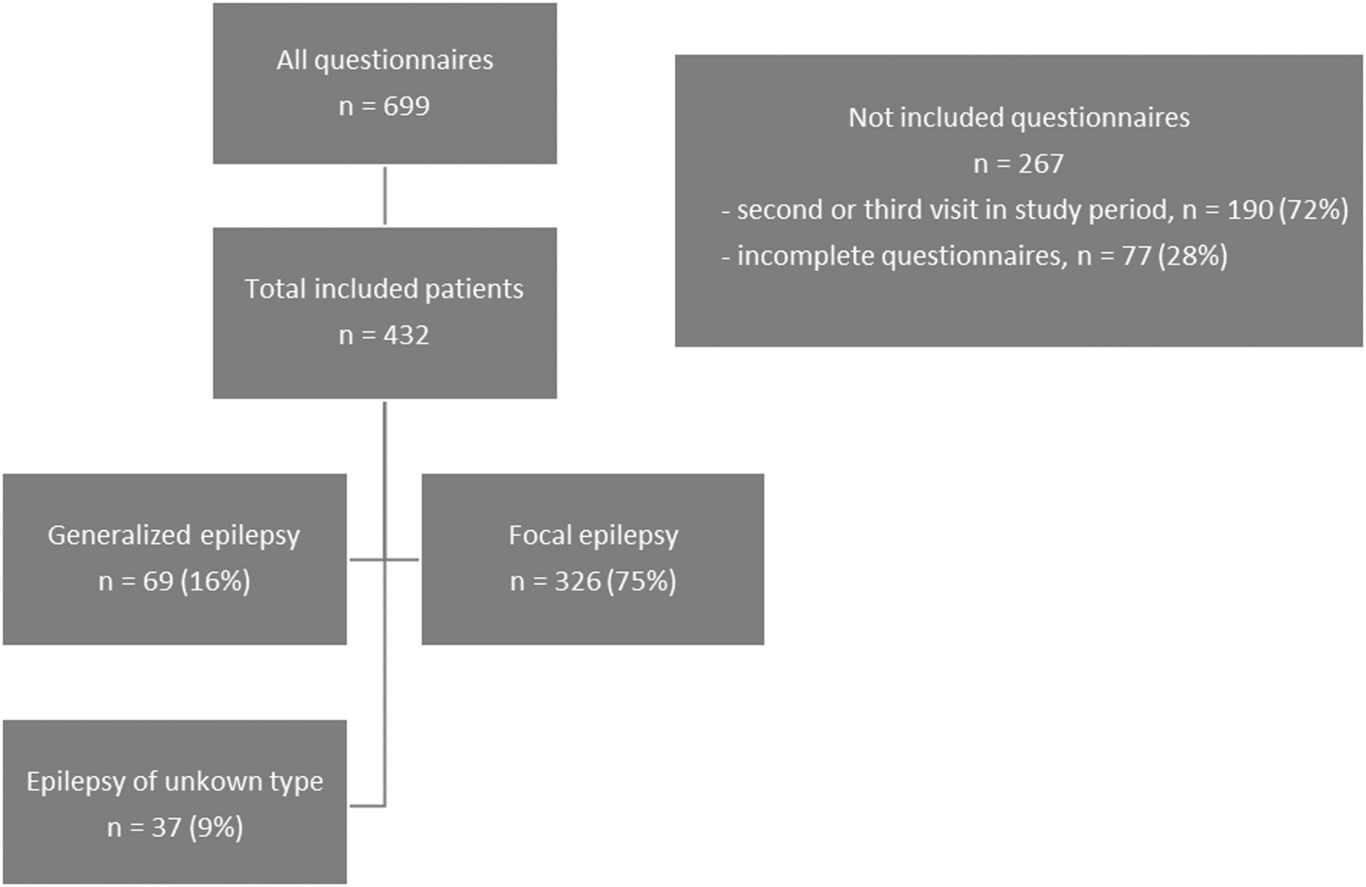
Stratification of the study population. A total of 699 sets of questionnaires were filled in. One set of questionnaires included the G‐LAEP, NDDI‐E and ET4. As only the questionnaires of the first visit during the study period were considered, 190 questionnaires were excluded as these were filled in second or third visits of the same patients. Seventy‐seven questionnaires were excluded due to incompletion. Ultimately, 432 sets of questionnaires and the same number of patients were included in this study. Sixty‐nine had generalized epilepsy, 326 had focal epilepsy and 37 had epilepsy of unknown type.

A total of 699 sets of questionnaires were filled in. One set of questionnaires included the LAEP, NDDI‐E and ET4. A total of 190 questionnaires were excluded as these were completed during second or third visits of the same patients and 77 questionnaires were excluded due to incompletion. Ultimately, 432 sets of questionnaires and the same number of patients were included in this study (Figure 1 – Stratification of the study population).

### 3.2 Demographic and clinical variables

Two hundred and twelve patients (49.7%) were of male gender. The median age was 42 (range = 19–90). Sixty‐nine respondents had generalized epilepsy (16%), 326 had focal epilepsy (75%) and 37 had epilepsy of unknown type (9%). A minimum of 12 months of seizure remission (at the time of completion of the questionnaires) was achieved by 134 patients (31%). All patients were on ASM therapy (one ASM, *n* = 213, 49.3%; two ASMs, *n* = 172, 39.8%; three ASMs, *n* = 47, 10.9%) with one or more of the following ASMs: levetiracetam, lamotrigine, valproate, carbamazepine, topiramate, lacosamide, perampanel, phenytoin, and/or gabapentin.

According to the LAEP 408 (94.4%) patients reported at least one AE. For every patient, “reporting at least one AE” is defined by choosing an answer other than “Never a problem” for at least one item. The most frequently reported AEs were “tiredness” (*n* = 333, 77.1%), “sleepiness” (*n* = 272, 62.9%) and “headache” (*n* = 268, 62%). A total of 98 patients reported a high total AE burden (22.7%).

A total of 72 patients were screened positive for depression (16.7%) according the NDDI‐E (cutoff > 13). A total of 59 patients were significantly impacted by “anxiety” (13.7%, cutoff > 5) and 49 patients by “anger” (11.3%, cutoff > 5) according to the ET.

Depression, total NDDI‐E score, ET anxiety score, ET anger score, number of ASMs, lack of seizure remission for at least 12 months and female gender correlated with higher LAEP scores.

Demographic and clinical variables including results of a univariate analysis are displayed in Table 1.

**TABLE 1:** Univariate analysis of demographic and clinical variables (*n* = 432).

	LAEP ≤ 44	LAEP > 44	2‐Tailed *p*‐value
	*n* = 334	*n* = 98	
Age in years, median (range)	40 (16–85)	45 (17–90)	n.s.
Gender, male (*n*)	176	36	Chi‐square = 7.72 *p* = 0.005
Epilepsy type			n.s.
Generalized (*n*)	64	5	
Focal (*n*)	252	74	
Epilepsy of unknown type (*n*)	22	15	
Seizure remission, 12 months (*n*)	116	18	Chi‐square = 9.48 *p* = 0.002
Number of ASMs, median (range)	1 (1–4)	2 (1–4)	*U* = 13 865 *p* = 0.011
Depression (=NDDI‐E > 13) (*n*)	27	45	Chi‐square = 78.09 *p* < 0.001
Total NDDI‐E score, median (range)	8 (5–19)	13 (6–20)	*U* = 5193 *p* < 0.001
ET anxiety score, median (range)	1 (0–10)	4 (0–10)	*U* = 8907 *p* < 0.001
ET anger score, median (range)	1 (0–9)	3 (0–10)	*U* = 10 095 *p* < 0.001
Total LAEP score, median (range)	30 (19–44)	49 (45–69)	

Abbreviations: ASM, antiseizure medication; ET, Emotional thermometer; LAEP, Liverpool Adverse Event Profile; NDDIE, Neurological Disorder Depression Inventory for Epilepsy.

### 3.3 Discriminant analysis of LAEP items and depression

The strongest discriminators for depression in descending order were the LAEP items “Depression”, “Nervousness/agitation”, “Tiredness,” and “Feelings of anger/aggression”.

The weakest discriminators in descending order were “Weight Gain”, “Hair loss”, “Double/blurred vision,” and “Problems with skin”. The results of the discriminant analysis are summarized in Table 2.

**TABLE 2:** Discriminant analysis of LAEP items and depression (NDDI‐E > 13).

LAEP items	Correlation coefficient
Depression	0.818
Nervousness/agitation	0.632
Tiredness	0.518
Feelings of anger/aggression	0.517
Memory problems	0.512
Difficulty in concentrating	0.492
Restlessness	0.488
Shaky hands	0.429
Unsteadiness	0.379
Disturbed sleep	0.372
Sleepiness	0.372
Trouble with mouth and gum	0.363
Upset stomach	0.335
Dizziness	0.316
Headache	0.293
Problems with skin	0.284
Double/blurred vision	0.283
Hair loss	0.205
Weight Gain	0.199

Abbreviation: LAEP, Liverpool Adverse Event Profile.

### 3.4 Logistic regression of LAEP items and depressive symptoms

**FIGURE 2:**
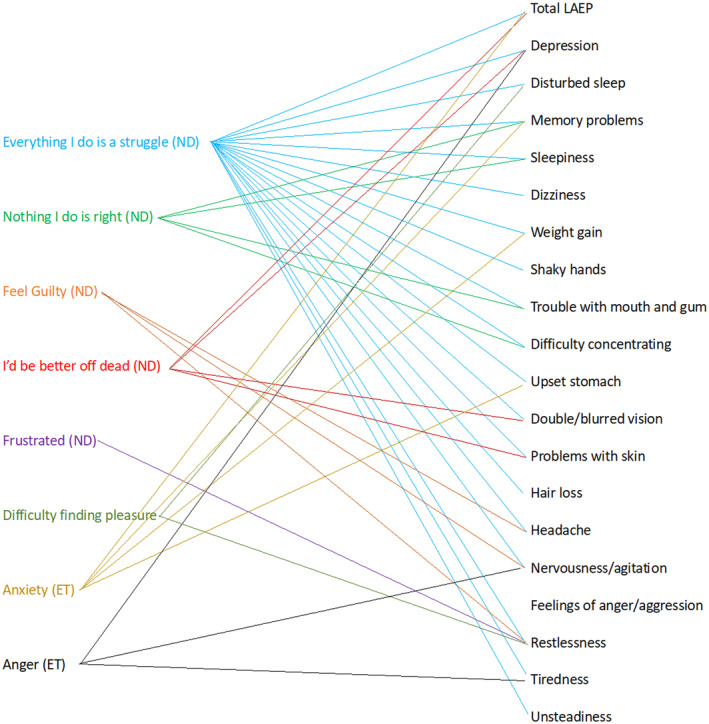
Correlation of depressive symptoms with high LAEP items. Each arrow demonstrates a significant correlation of a specific depressive symptom with the respective LAEP item reported as always present. ET, Emotional thermometer; LAEP, Liverpool Adverse Event Profile; ND, Neurological Disorder Depression Inventory for Epilepsy.

Out of all depressive symptoms “Everything I do is a struggle” most strongly correlated with total LAEP score (odds ratio [OR] = 3.1) and correlated with all LAEP items except “Feelings of anger/aggression”. “Nothing I do is right” correlated with “Memory problems” (OR = 1.6), “Sleepiness” (OR = 1.7) and “Difficulty concentrating” (OR = 1.5). “Feel guilty” correlated with “Trouble with mouth and gum” (OR = 1.5), “Headache” (OR = 1.6), “Nervousness/agitation” (1.6) and “Restlessness” (1.5). “I'd be better off dead” correlated with total LAEP score (OR = 2), “Depression” (OR = 2.1), “Double/blurred vision” (OR = 1.6) and “Problems with skin” (OR = 2.2). “Frustrated” correlated with “Restlessness” (OR = 1.5). “Difficulty finding pleasure” correlated with “Restlessness” (OR = 1.4) and “Disturbed sleep” (OR = 1.4). “Anxiety” correlated with total LAEP score (OR = 1.2), “Memory problems” (OR = 1.2), “Weight gain” (OR = 1.1) and “Difficulty concentrating” (OR = 1.2). “Anger” correlated with “Nervousness/agitation” (OR = 1.6) and “Feelings of anger/aggression” (OR = 1.5). All significant correlations are depicted in Figure 2. The results of the discriminant analysis are summarized in Table 3. Full data including *p*‐values and odds ratios can be found in the Supplementary Material section.

**TABLE 3:** Logistic regression of LAEP items and depressive symptoms.

	Total LAEP	Depression	Disturbed sleep	Memory problems	Sleepiness	Dizziness	Weight gain	Shaky hands	Trouble with mouth and gum	Difficulty concentrating	Upset stomach	Double/Blurred vision	Problems with skin	Hair loss	Headache	Nervousness/Agitation	Feelings of anger/Aggression	Restlessness	Tiredness	Unsteadiness
Everything I do is a struggle (ND)	3.1	1.5	1.8	1.6	1.9	1.5	1.7	1.5	1.3	1.5	2.1	1.4	1.7	1.5	1.6	1.4		1.6	3.4	2.7
Nothing I do is right (ND)				1.6	1.7					1.5										
Feel guilty (ND)									1.5						1.6	1.6		1.5		
I'd be better off dead (ND)	2	2.1										1.6	2.2							
Frustrated (ND)																				
Difficulty finding pleasure (ND)		2.4	1.4															1.4		
Anxiety (ET)	1.2			1.2			1.1			1.2										
Anger (ET)																1.6	1.5			

*Note*: Total LAEP and item scores are depicted column‐wise, while depressive symptoms are listed row‐wise. Independent variables including age, gender, epilepsy type, seizure remission and number of antiseizure medications were included. Odds ratios are only displayed if *p* < 0.05.

Abbreviations: ET, Emotional thermometer; LAEP, Liverpool Adverse Event Profile; ND, Neurological Disorder Depression Inventory for Epilepsy.

## 4 DISCUSSION

In this cross‐sectional study, we assessed the correlation of various depressive symptoms (=NDDI‐E and ET4 items) with specific AEs (=LAEP item scores) in PWE on ASM therapy.

We found that the presence of depression has a profound impact on both total LAEP and item scores, including both emotional and “somatic” symptoms. While it was not possible to determine cause‐and‐effect due to the nature of the study design the findings suggest a significant confounding effect of depression on the perception of an AE burden. Additionally, particular depressive symptoms correlated to varying degrees with specific LAEP items. This information may be helpful for clinical practice to quickly evaluate the likelihood of whether the reporting of specific LAEP items is confounded by particular depressive symptoms. This is relevant because the adaptation of therapy may differ depending on if AEs are solely caused by ASM therapy or are confounded by depressive symptoms. Simply reporting a particular depressive symptom may give a clue as to whether specific AEs are confounded by depression. Thus, we confirm the importance of screening for depression in all PWE. However, a formal psychiatric evaluation is still recommended and cannot be replaced by screening methods. An alternative is the NDDI‐E which is a validated self‐reported screening method for quick and easy detection of various depressive symptoms and major depressive disorder in PWE.^6^

In comparison to objective assessment by observers, more complaints are reported by PWE who evaluate themselves subjectively.^13^ Thus, using self‐reported questionnaires for the measurement of AEs in PWE might lead to overreporting of these complaints. A population‐based study using mail survey reported increased AEs in PWE with depression compared to those without.^14^ Determination of the cause‐and‐effect relationship is difficult as not only can depression increase perception of AEs, but high AE burden can reduce quality of life, compromise emotional well‐being, and thus, foster depression. This makes evaluation of AEs related to emotion especially difficult but as depression also causes “somatic” symptoms, assessment of the latter is affected as well.^15^

A significant impact of depression on LAEP scores has already been reported.^6^ However, previous studies analyzed the correlation of depression with total LAEP scores.^7^, ^16^ In contrast, to the best of our knowledge, we are the first to analyze the correlation of various depressive symptoms with specific LAEP items, where the aim is to assess which LAEP items are especially prone to being influenced by particular depressive symptoms.

A study using the Korean LAEP found that depression predicts items related to emotion and cognition but not “somatic” symptoms.^8^ In contrast, we found that depression and various depressive symptoms correlate with both items related and unrelated to emotion and cognition. This discrepancy may be the result of differences in the study design. Firstly, we studied the correlation of each LAEP item with depression, while the Korean study grouped LAEP items into different categories, which were then analyzed regarding their correlation with depression. Secondly, we studied the correlations of various depressive symptoms with specific LAEP items, while the Korean study only analyzed depression rather than individual symptoms.

Number of ASMs, lack of seizure remission for at least 12 months and female gender correlated with high total AE burden. Previous literature linked polytherapy using both newer and older ASMs with increased AEs and reduced quality of life.^17^, ^18^ Among others, common symptoms of polytherapy include tiredness, memory problems and difficulty concentrating.^17^ Other symptoms may be more specifically linked to individual ASMs such as the association of aggressiveness with levetiracetam.^19^ Lack of seizure freedom has also been linked to high total AE burden.^1^ This relationship may be explained by an on average higher number of ASMs and/or higher share of depression in the therapy‐refractory population. Female gender predicted several AEs. Women may be generally more prone to reporting AEs in pharmacotherapy. However, sex‐related variance may also depend on differences in genetics, physiology, immunology and hormonal balance.^20^

## 5 LIMITATIONS

Our findings are limited by the cross‐sectional study design and therefore the impossibility to determine causality. Psychiatric disorders may aggravate the perception of side effects and vice versa. However, the concept that psychiatric disorders confound both AEs related to emotion and somatic symptoms is well established in previous literature.

Depression was diagnosed by a self‐reported tool and not by the gold standard of psychiatric examination. However, the NDDI‐E has been previously validated as a reliable screening tool for major depressive disorder and various depressive symptoms in PWE.

As our patient group was selected from a specialized tertiary epilepsy service, we had a relatively high share of drug refractory PWE on polytherapy. Thus, different variables such as depression rates and distribution of AEs may not be representative for the general population of PWE.

## 6 CONCLUSION

Our results confirm a profound confounding effect of depression on total LAEP and item scores. We firstly studied the correlation of various depressive symptoms with specific LAEP items to understand which AEs are especially prone to being confounded by particular depressive symptoms. We found that different depressive symptoms correlate, to varying degrees, with specific LAEP items. Out of all depressive symptoms, the NDDI‐E item “Everything I do is a struggle” correlates most strongly with total LAEP and with the most item scores. In clinical practice, this information can be helpful for a fast evaluation of whether the reporting of specific LAEP items is confounded by particular depressive symptoms as shown in Figure 2. This is relevant because optimal adaption of therapy may differ depending on if AEs are confounded by depressive symptoms or are solely caused by ASM. Simply reporting a particular depressive symptom already provides a clue if specific AEs are confounded by depression. Thus, we confirm the importance of screening for depression in all PWE. However, formal psychiatric evaluation is still recommended and cannot be replaced by screening methods. Prospective studies are needed to explore the cause‐and‐effect relationship between depression and the AE burden and for evaluation of the potential benefit of depression therapy on the AE burden in PWE on ASM medication.

## AUTHOR CONTRIBUTIONS

TvO and JP designed the study. JP, AH, and NT performed the data acquisition. JP and TvO analyzed and interpreted the data. JP drafted the manuscript. TvO, GG, KY, AH, and NT revised the article with respect to intellectual content. All authors approved the final version of the manuscript for publication.

## FUNDING INFORMATION

This research received no specific grant from any funding agency in the public, commercial, or not‐for‐profit sectors.

## CONFLICT OF INTEREST STATEMENT

TvO received consulting fees or honoraria for lectures from Angelini Pharma, Arvelle, GW Pharma, Jazz Pharma, Liva Nova and Eisai, as well as travel and meeting support from Angelini pharma and Jazz Pharma, all outside of this study. All other authors declare that they do not maintain personal or financial relationships that present potential conflicts of interest. This research did not receive any specific grant from funding agencies in the public, commercial, or not‐for‐profit sectors. We confirm that we have read the Journal‘s position on issues involved in ethical publication and affirm that this report is consistent with those guidelines.

## ETHICS STATEMENT

Approval was obtained from the local ethics committee.

## PATIENT CONSENT STATEMENT

Written informed consent has been obtained from all patients.

## Supporting information


Supinfo

## Data Availability

Data will be provided by contacting JP.
