# Supplementary material for: Impact of depressive symptoms on adverse effects in people with epilepsy on antiseizure medication therapy
Source: Epilepsia Open. 2024 Apr 16;9(3):1067–75. doi: 10.1002/epi4.12943 (PMC11145617; doi:10.1002/epi4.12943)
Supplement: Supplementary file 1 — Supinfo [file EPI4-9-1067-s001.docx]

This document includes the complete information including p-values and odds ratios for all independent variables for all regression models in „Table 3 - Logistic regression of depressive symptoms and LAEP items“.

Abbreviations

- LAEP = Liverpool Adverse Events Profile
- ND = Neurological Disorders Depression Inventory for Epilepsy
- ET = Emotional Thermometers
- Remission 12 month = seizure remission for at least 12 months
- ASM = antiseizure medication
- Exp (b) = effect coefficient b

# Regression model for total LAEP score

|  | | | | | | | | | |
| --- | --- | --- | --- | --- | --- | --- | --- | --- | --- |
|  | | Coefficient B | Standard error | Wald | df | Sig. | Exp(B) | 95% Confidence interval for EXP(B) | |
|  |  |  |  |  |  |  |  | Lower value | Upper value |
| Schritt 1^a^ | ND_Struggle | 1,123 | ,179 | 39,468 | 1 | ,000 | 3,073 | 2,165 | 4,363 |
|  | ND_Wrong | ,015 | ,203 | ,005 | 1 | ,943 | 1,015 | ,682 | 1,509 |
|  | ND_Guilt | ,269 | ,199 | 1,830 | 1 | ,176 | 1,309 | ,886 | 1,935 |
|  | ND_Suicide | ,664 | ,294 | 5,092 | 1 | ,024 | 1,942 | 1,091 | 3,455 |
|  | ND_Frustration | -,050 | ,200 | ,062 | 1 | ,804 | ,952 | ,644 | 1,407 |
|  | ND_Joy | ,316 | ,181 | 3,058 | 1 | ,080 | 1,372 | ,963 | 1,955 |
|  | ET_Anxiety | ,151 | ,062 | 5,965 | 1 | ,015 | 1,163 | 1,030 | 1,313 |
|  | ET_Anger | ,001 | ,072 | ,000 | 1 | ,989 | 1,001 | ,869 | 1,153 |
|  | Age | ,004 | ,010 | ,198 | 1 | ,657 | 1,004 | ,986 | 1,023 |
|  | Gender | ,682 | ,317 | 4,642 | 1 | ,031 | 1,979 | 1,064 | 3,681 |
|  | Epilepsy type | ,021 | ,328 | ,004 | 1 | ,948 | 1,022 | ,537 | 1,943 |
|  | Remission 12 month | -,479 | ,359 | 1,777 | 1 | ,183 | ,620 | ,307 | 1,252 |
|  | ASM number | ,305 | ,216 | 1,995 | 1 | ,158 | 1,357 | ,888 | 2,073 |
|  | Constant | -7,222 | 1,075 | 45,096 | 1 | ,000 | ,001 |  |  |

|  | | | | | | | | | | |
| --- | --- | --- | --- | --- | --- | --- | --- | --- | --- | --- |
|  | | Coefficient B | Standard error | Wald | df | Sig. | Exp(B) | 95% Confidence interval for EXP(B) | |  |
|  |  |  |  |  |  |  |  | Lower value | Upper value |  |
| Schritt 1^a^ | ND_Struggle | ,434 | ,145 | 9,009 | 1 | ,003 | 1,543 | 1,163 | 2,049 |  |
|  | ND_Wrong | ,068 | ,189 | ,129 | 1 | ,720 | 1,070 | ,739 | 1,549 |  |
|  | ND_Guilt | -,006 | ,198 | ,001 | 1 | ,975 | ,994 | ,675 | 1,464 |  |
|  | ND_Suicide | ,738 | ,339 | 4,731 | 1 | ,030 | 2,091 | 1,076 | 4,065 |  |
|  | ND_Frustration | ,256 | ,185 | 1,916 | 1 | ,166 | 1,292 | ,899 | 1,856 |  |
|  | ND_Joy | ,857 | ,177 | 23,348 | 1 | ,000 | 2,356 | 1,664 | 3,336 |  |
|  | ET_Anxiety | ,086 | ,059 | 2,107 | 1 | ,147 | 1,090 | ,970 | 1,223 |  |
|  | ET_Anger | ,064 | ,069 | ,871 | 1 | ,351 | 1,067 | ,932 | 1,221 |  |
|  | Age | ,024 | ,008 | 9,081 | 1 | ,003 | 1,025 | 1,009 | 1,041 |  |
|  | Gender | ,254 | ,263 | ,931 | 1 | ,335 | 1,289 | ,769 | 2,160 |  |
|  | Epilepsy type | ,382 | ,274 | 1,944 | 1 | ,163 | 1,465 | ,856 | 2,506 |  |
|  | Remission 12 month | -,478 | ,294 | 2,649 | 1 | ,104 | ,620 | ,348 | 1,103 |  |
|  | ASM number | ,127 | ,195 | ,424 | 1 | ,515 | 1,135 | ,775 | 1,662 |  |
|  | Constant | -6,597 | ,946 | 48,610 | 1 | ,000 | ,001 |  |  |  |

# Regression model for LAEP item „Depression“

# Regression model for LAEP item „Disturbed sleep“

|  | | Coefficient B | Standard error | Wald | df | Sig. | Exp(B) | 95% Confidence interval for EXP(B) | |
| --- | --- | --- | --- | --- | --- | --- | --- | --- | --- |
|  |  |  |  |  |  |  |  | Lower value | Upper value |
| Schritt 1^a^ | ND_Struggle | ,605 | ,127 | 22,834 | 1 | ,000 | 1,832 | 1,429 | 2,348 |
|  | ND_Wrong | ,236 | ,167 | 1,999 | 1 | ,157 | 1,267 | ,913 | 1,758 |
|  | ND_Guilt | ,035 | ,173 | ,041 | 1 | ,840 | 1,036 | ,738 | 1,453 |
|  | ND_Suicide | -,077 | ,261 | ,086 | 1 | ,769 | ,926 | ,555 | 1,545 |
|  | ND_Frustration | ,040 | ,170 | ,056 | 1 | ,814 | 1,041 | ,746 | 1,451 |
|  | ND_Joy | ,349 | ,160 | 4,747 | 1 | ,029 | 1,418 | 1,036 | 1,941 |
|  | ET_Anxiety | -,033 | ,053 | ,393 | 1 | ,531 | ,967 | ,872 | 1,073 |
|  | ET_Anger | ,031 | ,061 | ,263 | 1 | ,608 | 1,032 | ,915 | 1,164 |
|  | Age | ,012 | ,007 | 3,010 | 1 | ,083 | 1,012 | ,998 | 1,025 |
|  | Gender | ,181 | ,219 | ,679 | 1 | ,410 | 1,198 | ,780 | 1,841 |
|  | Epilepsy type | ,030 | ,222 | ,018 | 1 | ,893 | 1,030 | ,667 | 1,592 |
|  | Remission 12 month | ,250 | ,241 | 1,077 | 1 | ,299 | 1,284 | ,801 | 2,057 |
|  | ASM number | ,077 | ,163 | ,223 | 1 | ,637 | 1,080 | ,784 | 1,488 |
|  | Constant | -3,053 | ,715 | 18,233 | 1 | ,000 | ,047 |  |  |

# Regression model for LAEP item „Memory problems“

|  | | | | | | | | | |
| --- | --- | --- | --- | --- | --- | --- | --- | --- | --- |
|  | | Coefficient B | Standard error | Wald | df | Sig. | Exp(B) | 95% Confidence interval for EXP(B) | |
|  |  |  |  |  |  |  |  | Lower value | Upper value |
| Schritt 1^a^ | ND_Struggle | ,467 | ,141 | 11,003 | 1 | ,001 | 1,595 | 1,210 | 2,101 |
|  | ND_Wrong | ,472 | ,196 | 5,761 | 1 | ,016 | 1,602 | 1,090 | 2,355 |
|  | ND_Guilt | -,175 | ,199 | ,772 | 1 | ,380 | ,840 | ,569 | 1,240 |
|  | ND_Suicide | ,515 | ,368 | 1,953 | 1 | ,162 | 1,673 | ,813 | 3,443 |
|  | ND_Frustration | ,158 | ,192 | ,680 | 1 | ,410 | 1,171 | ,804 | 1,705 |
|  | ND_Joy | ,052 | ,183 | ,081 | 1 | ,776 | 1,054 | ,736 | 1,508 |
|  | ET_Anxiety | ,145 | ,063 | 5,373 | 1 | ,020 | 1,157 | 1,023 | 1,308 |
|  | ET_Anger | ,055 | ,070 | ,613 | 1 | ,434 | 1,056 | ,921 | 1,211 |
|  | Age | ,036 | ,008 | 23,124 | 1 | ,000 | 1,037 | 1,022 | 1,053 |
|  | Gender | ,259 | ,241 | 1,156 | 1 | ,282 | 1,296 | ,808 | 2,077 |
|  | Epilepsy type | ,234 | ,243 | ,930 | 1 | ,335 | 1,264 | ,785 | 2,035 |
|  | Remission 12 month | -,476 | ,258 | 3,406 | 1 | ,065 | ,622 | ,375 | 1,030 |
|  | ASM number | ,312 | ,185 | 2,855 | 1 | ,091 | 1,366 | ,951 | 1,962 |
|  | Constant | -4,816 | ,848 | 32,235 | 1 | ,000 | ,008 |  |  |

# Regression model for LAEP item „Sleepiness“

|  | | | | | | | | | |
| --- | --- | --- | --- | --- | --- | --- | --- | --- | --- |
|  | | Coefficient B | Standard error | Wald | df | Sig. | Exp(B) | 95% Confidence interval for EXP(B) | |
|  |  |  |  |  |  |  |  | Lower value | Upper value |
| Schritt 1^a^ | ND_Struggle | ,656 | ,142 | 21,340 | 1 | ,000 | 1,926 | 1,459 | 2,544 |
|  | ND_Wrong | ,519 | ,199 | 6,806 | 1 | ,009 | 1,680 | 1,138 | 2,480 |
|  | ND_Guilt | -,100 | ,202 | ,245 | 1 | ,620 | ,905 | ,608 | 1,345 |
|  | ND_Suicide | ,142 | ,341 | ,173 | 1 | ,678 | 1,152 | ,591 | 2,246 |
|  | ND_Frustration | ,343 | ,198 | 3,006 | 1 | ,083 | 1,409 | ,956 | 2,076 |
|  | ND_Joy | -,134 | ,187 | ,519 | 1 | ,471 | ,874 | ,606 | 1,260 |
|  | ET_Anxiety | ,039 | ,061 | ,397 | 1 | ,529 | 1,039 | ,922 | 1,172 |
|  | ET_Anger | ,034 | ,069 | ,234 | 1 | ,628 | 1,034 | ,903 | 1,185 |
|  | Age | ,002 | ,007 | ,047 | 1 | ,829 | 1,002 | ,988 | 1,015 |
|  | Gender | -,213 | ,234 | ,826 | 1 | ,364 | ,809 | ,511 | 1,279 |
|  | Epilepsy type | ,100 | ,236 | ,181 | 1 | ,671 | 1,106 | ,696 | 1,757 |
|  | Remission 12 month | -,751 | ,248 | 9,181 | 1 | ,002 | ,472 | ,290 | ,767 |
|  | ASM number | -,025 | ,180 | ,019 | 1 | ,891 | ,976 | ,686 | 1,387 |
|  | Constant | -1,946 | ,764 | 6,485 | 1 | ,011 | ,143 |  |  |

# Regression model for LAEP item „Dizziness“

|  | | | | | | | | | | |
| --- | --- | --- | --- | --- | --- | --- | --- | --- | --- | --- |
|  | | Coefficient B | Standard error | Wald | df | Sig. | Exp(B) | 95% Confidence interval for EXP(B) | |  |
|  |  |  |  |  |  |  |  | Lower value | Upper value |  |
| Schritt 1^a^ | ND_Struggle | ,408 | ,129 | 10,086 | 1 | ,001 | 1,504 | 1,169 | 1,935 |  |
|  | ND_Wrong | ,256 | ,172 | 2,208 | 1 | ,137 | 1,291 | ,922 | 1,810 |  |
|  | ND_Guilt | ,032 | ,182 | ,032 | 1 | ,859 | 1,033 | ,723 | 1,475 |  |
|  | ND_Suicide | -,066 | ,283 | ,054 | 1 | ,817 | ,936 | ,538 | 1,631 |  |
|  | ND_Frustration | ,266 | ,174 | 2,332 | 1 | ,127 | 1,305 | ,927 | 1,835 |  |
|  | ND_Joy | -,075 | ,164 | ,207 | 1 | ,649 | ,928 | ,673 | 1,280 |  |
|  | ET_Anxiety | ,093 | ,056 | 2,763 | 1 | ,096 | 1,098 | ,983 | 1,225 |  |
|  | ET_Anger | ,134 | ,063 | 4,525 | 1 | ,033 | 1,143 | 1,011 | 1,294 |  |
|  | Age | ,020 | ,007 | 8,500 | 1 | ,004 | 1,021 | 1,007 | 1,035 |  |
|  | Gender | ,476 | ,229 | 4,310 | 1 | ,038 | 1,610 | 1,027 | 2,524 |  |
|  | Epilepsy type | ,549 | ,239 | 5,295 | 1 | ,021 | 1,732 | 1,085 | 2,766 |  |
|  | Remission 12 month | -,270 | ,252 | 1,148 | 1 | ,284 | ,763 | ,466 | 1,251 |  |
|  | ASM number | ,287 | ,172 | 2,776 | 1 | ,096 | 1,332 | ,951 | 1,867 |  |
|  | Constant | -4,584 | ,791 | 33,547 | 1 | ,000 | ,010 |  |  |  |

# Regression model for LAEP item „Weight gain“

|  | | | | | | | | | |
| --- | --- | --- | --- | --- | --- | --- | --- | --- | --- |
|  | | Coefficient B | Standard error | Wald | df | Sig. | Exp(B) | 95% Confidence interval for EXP(B) | |
|  |  |  |  |  |  |  |  | Lower value | Upper value |
| Schritt 1^a^ | ND_Struggle | ,527 | ,127 | 17,262 | 1 | ,000 | 1,694 | 1,321 | 2,172 |
|  | ND_Wrong | ,014 | ,166 | ,007 | 1 | ,932 | 1,014 | ,733 | 1,404 |
|  | ND_Guilt | -,003 | ,167 | ,000 | 1 | ,988 | ,997 | ,720 | 1,383 |
|  | ND_Suicide | -,008 | ,245 | ,001 | 1 | ,974 | ,992 | ,614 | 1,602 |
|  | ND_Frustration | ,151 | ,163 | ,863 | 1 | ,353 | 1,163 | ,845 | 1,601 |
|  | ND_Joy | -,094 | ,154 | ,376 | 1 | ,540 | ,910 | ,673 | 1,230 |
|  | ET_Anxiety | ,117 | ,051 | 5,324 | 1 | ,021 | 1,125 | 1,018 | 1,242 |
|  | ET_Anger | -,060 | ,059 | 1,049 | 1 | ,306 | ,942 | ,840 | 1,056 |
|  | Age | ,007 | ,007 | 1,070 | 1 | ,301 | 1,007 | ,994 | 1,021 |
|  | Gender | ,178 | ,225 | ,628 | 1 | ,428 | 1,195 | ,769 | 1,857 |
|  | Epilepsy type | -,048 | ,232 | ,044 | 1 | ,834 | ,953 | ,605 | 1,500 |
|  | Remission 12 month | ,546 | ,247 | 4,901 | 1 | ,027 | 1,727 | 1,065 | 2,801 |
|  | ASM number | ,251 | ,164 | 2,363 | 1 | ,124 | 1,286 | ,933 | 1,771 |
|  | Constant | -2,982 | ,712 | 17,568 | 1 | ,000 | ,051 |  |  |

# Regression model for LAEP item „Shaky hands“

|  | | Coefficient B | Standard error | Wald | df | Sig. | Exp(B) | 95% Confidence interval for EXP(B) | |
| --- | --- | --- | --- | --- | --- | --- | --- | --- | --- |
|  |  |  |  |  |  |  |  | Lower value | Upper value |
| Schritt 1^a^ | ND_Struggle | ,381 | ,126 | 9,187 | 1 | ,002 | 1,463 | 1,144 | 1,871 |
|  | ND_Wrong | ,254 | ,166 | 2,331 | 1 | ,127 | 1,289 | ,930 | 1,787 |
|  | ND_Guilt | ,118 | ,172 | ,476 | 1 | ,490 | 1,126 | ,804 | 1,576 |
|  | ND_Suicide | ,255 | ,283 | ,814 | 1 | ,367 | 1,290 | ,742 | 2,245 |
|  | ND_Frustration | ,080 | ,169 | ,226 | 1 | ,635 | 1,084 | ,778 | 1,509 |
|  | ND_Joy | ,031 | ,157 | ,038 | 1 | ,845 | 1,031 | ,758 | 1,403 |
|  | ET_Anxiety | -,006 | ,053 | ,011 | 1 | ,917 | ,995 | ,897 | 1,103 |
|  | ET_Anger | ,086 | ,061 | 2,006 | 1 | ,157 | 1,090 | ,968 | 1,227 |
|  | Age | ,019 | ,007 | 8,149 | 1 | ,004 | 1,020 | 1,006 | 1,033 |
|  | Gender | ,001 | ,222 | ,000 | 1 | ,996 | 1,001 | ,648 | 1,547 |
|  | Epilepsy type | -,032 | ,227 | ,020 | 1 | ,888 | ,969 | ,621 | 1,512 |
|  | Remission 12 month | -,367 | ,242 | 2,293 | 1 | ,130 | ,693 | ,431 | 1,114 |
|  | ASM number | ,577 | ,168 | 11,805 | 1 | ,001 | 1,781 | 1,281 | 2,474 |
|  | Constant | -3,644 | ,736 | 24,496 | 1 | ,000 | ,026 |  |  |

|  | | Coefficient B | Standard error | Wald | df | Sig. | Exp(B) | 95% Confidence interval for EXP(B) | |
| --- | --- | --- | --- | --- | --- | --- | --- | --- | --- |
|  |  |  |  |  |  |  |  | Lower value | Upper value |
| Schritt 1^a^ | ND_Struggle | ,286 | ,126 | 5,150 | 1 | ,023 | 1,332 | 1,040 | 1,705 |
|  | ND_Wrong | ,238 | ,163 | 2,124 | 1 | ,145 | 1,269 | ,921 | 1,748 |
|  | ND_Guilt | ,414 | ,168 | 6,106 | 1 | ,013 | 1,513 | 1,089 | 2,101 |
|  | ND_Suicide | ,170 | ,251 | ,460 | 1 | ,498 | 1,185 | ,725 | 1,938 |
|  | ND_Frustration | ,130 | ,165 | ,620 | 1 | ,431 | 1,139 | ,824 | 1,574 |
|  | ND_Joy | -,125 | ,156 | ,650 | 1 | ,420 | ,882 | ,650 | 1,197 |
|  | ET_Anxiety | ,082 | ,052 | 2,526 | 1 | ,112 | 1,085 | ,981 | 1,201 |
|  | ET_Anger | ,003 | ,059 | ,002 | 1 | ,963 | 1,003 | ,894 | 1,125 |
|  | Age | -,004 | ,007 | ,341 | 1 | ,559 | ,996 | ,982 | 1,010 |
|  | Gender | ,132 | ,227 | ,338 | 1 | ,561 | 1,141 | ,731 | 1,782 |
|  | Epilepsy type | ,143 | ,230 | ,385 | 1 | ,535 | 1,154 | ,735 | 1,812 |
|  | Remission 12 month | ,669 | ,250 | 7,159 | 1 | ,007 | 1,952 | 1,196 | 3,187 |
|  | ASM number | ,290 | ,167 | 3,039 | 1 | ,081 | 1,337 | ,965 | 1,853 |
|  | Constant | -3,559 | ,733 | 23,563 | 1 | ,000 | ,028 |  |  |

# Regression model for LAEP item „Trouble with mouth and gum“

# Regression model for LAEP item „Difficulty concentrating“

|  | | | | | | | | | | |
| --- | --- | --- | --- | --- | --- | --- | --- | --- | --- | --- |
|  | | Coefficient B | Standard error | Wald | df | Sig. | Exp(B) | 95% Confidence interval for EXP(B) | |  |
|  |  |  |  |  |  |  |  | Lower value | Upper value |  |
| Schritt 1^a^ | ND_Struggle | ,406 | ,137 | 8,749 | 1 | ,003 | 1,501 | 1,147 | 1,964 |  |
|  | ND_Wrong | ,405 | ,194 | 4,362 | 1 | ,037 | 1,500 | 1,025 | 2,194 |  |
|  | ND_Guilt | ,152 | ,204 | ,553 | 1 | ,457 | 1,164 | ,780 | 1,737 |  |
|  | ND_Suicide | ,570 | ,420 | 1,844 | 1 | ,174 | 1,768 | ,777 | 4,024 |  |
|  | ND_Frustration | ,150 | ,194 | ,597 | 1 | ,440 | 1,162 | ,794 | 1,701 |  |
|  | ND_Joy | -,008 | ,186 | ,002 | 1 | ,967 | ,992 | ,690 | 1,428 |  |
|  | ET_Anxiety | ,172 | ,066 | 6,793 | 1 | ,009 | 1,187 | 1,043 | 1,351 |  |
|  | ET_Anger | ,067 | ,071 | ,886 | 1 | ,347 | 1,069 | ,930 | 1,229 |  |
|  | Age | ,002 | ,007 | ,113 | 1 | ,736 | 1,002 | ,989 | 1,016 |  |
|  | Gender | ,206 | ,234 | ,773 | 1 | ,379 | 1,229 | ,776 | 1,944 |  |
|  | Epilepsy type | ,213 | ,239 | ,793 | 1 | ,373 | 1,237 | ,774 | 1,977 |  |
|  | Remission 12 month | -,655 | ,252 | 6,741 | 1 | ,009 | ,520 | ,317 | ,852 |  |
|  | ASM number | ,248 | ,182 | 1,854 | 1 | ,173 | 1,281 | ,897 | 1,831 |  |
|  | Constant | -3,331 | ,821 | 16,456 | 1 | ,000 | ,036 |  |  |  |
|  | | | | | | | | | | |

|  | | | | | | | | | | |
| --- | --- | --- | --- | --- | --- | --- | --- | --- | --- | --- |
|  | | Coefficient B | Standard error | Wald | df | Sig. | Exp(B) | 95% Confidence interval for EXP(B) | |  |
|  |  |  |  |  |  |  |  | Lower value | Upper value |  |
| Schritt 1^a^ | ND_Struggle | ,760 | ,133 | 32,732 | 1 | ,000 | 2,138 | 1,648 | 2,773 |  |
|  | ND_Wrong | -,048 | ,169 | ,082 | 1 | ,775 | ,953 | ,684 | 1,327 |  |
|  | ND_Guilt | ,205 | ,169 | 1,472 | 1 | ,225 | 1,227 | ,882 | 1,709 |  |
|  | ND_Suicide | ,027 | ,247 | ,012 | 1 | ,913 | 1,027 | ,633 | 1,666 |  |
|  | ND_Frustration | ,116 | ,166 | ,490 | 1 | ,484 | 1,123 | ,811 | 1,556 |  |
|  | ND_Joy | -,066 | ,157 | ,178 | 1 | ,673 | ,936 | ,689 | 1,272 |  |
|  | ET_Anxiety | ,003 | ,052 | ,004 | 1 | ,951 | 1,003 | ,906 | 1,111 |  |
|  | ET_Anger | ,010 | ,060 | ,025 | 1 | ,873 | 1,010 | ,898 | 1,135 |  |
|  | Age | -,012 | ,007 | 2,614 | 1 | ,106 | ,988 | ,975 | 1,002 |  |
|  | Gender | ,334 | ,230 | 2,100 | 1 | ,147 | 1,396 | ,889 | 2,193 |  |
|  | Epilepsy type | ,197 | ,235 | ,704 | 1 | ,401 | 1,218 | ,768 | 1,932 |  |
|  | Remission 12 month | ,288 | ,253 | 1,292 | 1 | ,256 | 1,334 | ,812 | 2,192 |  |
|  | ASM number | ,171 | ,168 | 1,039 | 1 | ,308 | 1,187 | ,854 | 1,649 |  |
|  | Constant | -3,198 | ,734 | 18,997 | 1 | ,000 | ,041 |  |  |  |
|  | | | | | | | | | | |

# Regression model for LAEP item „Upset stomach“

# Regression model for LAEP item „Double/blurred vision“

|  | | | | | | | | | |
| --- | --- | --- | --- | --- | --- | --- | --- | --- | --- |
|  | | Coefficient B | Standard error | Wald | df | Sig. | Exp(B) | 95% Confidence interval for EXP(B) | |
|  |  |  |  |  |  |  |  | Lower value | Upper value |
| Schritt 1^a^ | ND_Struggle | ,366 | ,127 | 8,295 | 1 | ,004 | 1,442 | 1,124 | 1,850 |
|  | ND_Wrong | -,022 | ,167 | ,017 | 1 | ,896 | ,978 | ,706 | 1,357 |
|  | ND_Guilt | -,003 | ,169 | ,000 | 1 | ,984 | ,997 | ,715 | 1,389 |
|  | ND_Suicide | ,508 | ,252 | 4,062 | 1 | ,044 | 1,662 | 1,014 | 2,724 |
|  | ND_Frustration | ,178 | ,164 | 1,172 | 1 | ,279 | 1,195 | ,866 | 1,649 |
|  | ND_Joy | -,057 | ,153 | ,137 | 1 | ,711 | ,945 | ,700 | 1,276 |
|  | ET_Anxiety | ,038 | ,051 | ,569 | 1 | ,451 | 1,039 | ,941 | 1,147 |
|  | ET_Anger | ,035 | ,058 | ,366 | 1 | ,545 | 1,036 | ,924 | 1,162 |
|  | Age | ,007 | ,007 | ,930 | 1 | ,335 | 1,007 | ,993 | 1,021 |
|  | Gender | ,080 | ,230 | ,120 | 1 | ,729 | 1,083 | ,690 | 1,701 |
|  | Epilepsy type | ,576 | ,246 | 5,482 | 1 | ,019 | 1,779 | 1,098 | 2,881 |
|  | Remission 12 month | -,058 | ,258 | ,052 | 1 | ,820 | ,943 | ,569 | 1,562 |
|  | ASM number | ,431 | ,166 | 6,709 | 1 | ,010 | 1,539 | 1,111 | 2,133 |
|  | Constant | -4,668 | ,783 | 35,558 | 1 | ,000 | ,009 |  |  |
|  | | | | | | | | | |

# Regression model for LAEP item „Problems with skin“

|  | | | | | | | | | |
| --- | --- | --- | --- | --- | --- | --- | --- | --- | --- |
|  | | Coefficient B | Standard error | Wald | df | Sig. | Exp(B) | 95% Confidence interval for EXP(B) | |
|  |  |  |  |  |  |  |  | Lower value | Upper value |
| Schritt 1^a^ | ND_Struggle | ,500 | ,132 | 14,324 | 1 | ,000 | 1,649 | 1,273 | 2,136 |
|  | ND_Wrong | -,096 | ,174 | ,305 | 1 | ,581 | ,908 | ,646 | 1,278 |
|  | ND_Guilt | -,053 | ,175 | ,094 | 1 | ,759 | ,948 | ,673 | 1,335 |
|  | ND_Suicide | ,793 | ,267 | 8,813 | 1 | ,003 | 2,211 | 1,309 | 3,732 |
|  | ND_Frustration | -,008 | ,167 | ,002 | 1 | ,963 | ,992 | ,715 | 1,377 |
|  | ND_Joy | ,032 | ,159 | ,040 | 1 | ,842 | 1,032 | ,756 | 1,410 |
|  | ET_Anxiety | -,003 | ,052 | ,004 | 1 | ,947 | ,997 | ,899 | 1,104 |
|  | ET_Anger | ,075 | ,059 | 1,602 | 1 | ,206 | 1,077 | ,960 | 1,209 |
|  | Age | -,028 | ,008 | 13,345 | 1 | ,000 | ,973 | ,958 | ,987 |
|  | Gender | ,082 | ,233 | ,124 | 1 | ,725 | 1,085 | ,688 | 1,713 |
|  | Epilepsy type | ,228 | ,233 | ,954 | 1 | ,329 | 1,256 | ,795 | 1,985 |
|  | Remission 12 month | -,070 | ,257 | ,074 | 1 | ,785 | ,932 | ,563 | 1,543 |
|  | ASM number | ,133 | ,169 | ,616 | 1 | ,432 | 1,142 | ,819 | 1,592 |
|  | Constant | -2,313 | ,726 | 10,151 | 1 | ,001 | ,099 |  |  |

# Regression model for LAEP item „Hair loss“

|  | | | | | | | | | |
| --- | --- | --- | --- | --- | --- | --- | --- | --- | --- |
|  | | Coefficient B | Standard error | Wald | df | Sig. | Exp(B) | 95% Confidence interval for EXP(B) | |
|  |  |  |  |  |  |  |  | Lower value | Upper value |
| Schritt 1^a^ | ND_Struggle | ,409 | ,134 | 9,344 | 1 | ,002 | 1,505 | 1,158 | 1,956 |
|  | ND_Wrong | ,066 | ,172 | ,146 | 1 | ,702 | 1,068 | ,763 | 1,496 |
|  | ND_Guilt | -,019 | ,174 | ,012 | 1 | ,912 | ,981 | ,697 | 1,380 |
|  | ND_Suicide | ,325 | ,246 | 1,748 | 1 | ,186 | 1,384 | ,855 | 2,240 |
|  | ND_Frustration | ,164 | ,171 | ,920 | 1 | ,338 | 1,178 | ,843 | 1,646 |
|  | ND_Joy | -,019 | ,162 | ,013 | 1 | ,909 | ,982 | ,715 | 1,348 |
|  | ET_Anxiety | -,003 | ,055 | ,004 | 1 | ,951 | ,997 | ,896 | 1,109 |
|  | ET_Anger | ,021 | ,061 | ,126 | 1 | ,723 | 1,022 | ,907 | 1,150 |
|  | Age | ,008 | ,007 | 1,272 | 1 | ,259 | 1,008 | ,994 | 1,023 |
|  | Gender | ,972 | ,248 | 15,413 | 1 | ,000 | 2,643 | 1,627 | 4,295 |
|  | Epilepsy type | -,023 | ,253 | ,008 | 1 | ,928 | ,977 | ,595 | 1,604 |
|  | Remission 12 month | ,132 | ,266 | ,247 | 1 | ,619 | 1,141 | ,678 | 1,921 |
|  | ASM number | -,009 | ,175 | ,003 | 1 | ,957 | ,991 | ,703 | 1,395 |
|  | Constant | -3,611 | ,767 | 22,163 | 1 | ,000 | ,027 |  |  |
|  | | | | | | | | | |

# Regression model for LAEP item „Headache“

|  | | | | | | | | | |
| --- | --- | --- | --- | --- | --- | --- | --- | --- | --- |
|  | | Coefficient B | Standard error | Wald | df | Sig. | Exp(B) | 95% Confidence interval for EXP(B) | |
|  |  |  |  |  |  |  |  | Lower value | Upper value |
| Schritt 1^a^ | ND_Struggle | ,443 | ,131 | 11,468 | 1 | ,001 | 1,558 | 1,205 | 2,013 |
|  | ND_Wrong | ,023 | ,178 | ,016 | 1 | ,899 | 1,023 | ,721 | 1,451 |
|  | ND_Guilt | ,476 | ,198 | 5,756 | 1 | ,016 | 1,609 | 1,091 | 2,373 |
|  | ND_Suicide | ,426 | ,315 | 1,825 | 1 | ,177 | 1,530 | ,825 | 2,837 |
|  | ND_Frustration | -,264 | ,178 | 2,185 | 1 | ,139 | ,768 | ,542 | 1,090 |
|  | ND_Joy | ,170 | ,167 | 1,038 | 1 | ,308 | 1,185 | ,855 | 1,643 |
|  | ET_Anxiety | ,019 | ,056 | ,117 | 1 | ,732 | 1,019 | ,913 | 1,138 |
|  | ET_Anger | ,037 | ,065 | ,320 | 1 | ,572 | 1,038 | ,913 | 1,179 |
|  | Age | -,013 | ,007 | 3,869 | 1 | ,049 | ,987 | ,974 | 1,000 |
|  | Gender | ,844 | ,221 | 14,631 | 1 | ,000 | 2,326 | 1,509 | 3,586 |
|  | Epilepsy type | ,187 | ,227 | ,682 | 1 | ,409 | 1,206 | ,773 | 1,881 |
|  | Remission 12 month | ,072 | ,243 | ,087 | 1 | ,768 | 1,074 | ,667 | 1,730 |
|  | ASM number | ,052 | ,166 | ,098 | 1 | ,755 | 1,053 | ,761 | 1,458 |
|  | Constant | -1,885 | ,723 | 6,805 | 1 | ,009 | ,152 |  |  |
|  | | | | | | | | | |

|  | | Coefficient B | Standard error | Wald | df | Sig. | Exp(B) | 95% Confidence interval for EXP(B) | |
| --- | --- | --- | --- | --- | --- | --- | --- | --- | --- |
|  |  |  |  |  |  |  |  | Lower value | Upper value |
| Schritt 1^a^ | ND_Struggle | ,333 | ,135 | 6,106 | 1 | ,013 | 1,395 | 1,071 | 1,816 |
|  | ND_Wrong | -,314 | ,191 | 2,701 | 1 | ,100 | ,731 | ,503 | 1,062 |
|  | ND_Guilt | ,478 | ,193 | 6,152 | 1 | ,013 | 1,613 | 1,106 | 2,355 |
|  | ND_Suicide | ,313 | ,360 | ,757 | 1 | ,384 | 1,367 | ,676 | 2,766 |
|  | ND_Frustration | ,207 | ,182 | 1,293 | 1 | ,255 | 1,229 | ,861 | 1,755 |
|  | ND_Joy | ,043 | ,174 | ,060 | 1 | ,806 | 1,044 | ,742 | 1,468 |
|  | ET_Anxiety | ,065 | ,058 | 1,263 | 1 | ,261 | 1,068 | ,952 | 1,197 |
|  | ET_Anger | ,405 | ,073 | 30,428 | 1 | ,000 | 1,499 | 1,298 | 1,731 |
|  | Age | -,004 | ,007 | ,283 | 1 | ,595 | ,996 | ,982 | 1,010 |
|  | Gender | -,069 | ,237 | ,086 | 1 | ,770 | ,933 | ,586 | 1,484 |
|  | Epilepsy type | ,447 | ,243 | 3,393 | 1 | ,065 | 1,563 | ,972 | 2,514 |
|  | Remission 12 month | -,218 | ,262 | ,694 | 1 | ,405 | ,804 | ,481 | 1,344 |
|  | ASM number | -,042 | ,180 | ,053 | 1 | ,818 | ,959 | ,674 | 1,365 |
|  | Constant | -3,144 | ,798 | 15,517 | 1 | ,000 | ,043 |  |  |
|  | | | | | | | | | |

# Regression model for LAEP item „Nervousness/agitation“

# Regression model for LAEP item „Feelings of anger/aggression“

|  | | Coefficient B | Standard error | Wald | df | Sig. | Exp(B) | 95% Confidence interval for EXP(B) | |  |
| --- | --- | --- | --- | --- | --- | --- | --- | --- | --- | --- |
|  |  |  |  |  |  |  |  | Lower value | Upper value |  |
|  | ND_Struggle | ,165 | ,137 | 1,456 | 1 | ,228 | 1,179 | ,902 | 1,542 |  |
|  | ND_Wrong | ,140 | ,179 | ,611 | 1 | ,434 | 1,150 | ,810 | 1,634 |  |
|  | ND_Guilt | ,281 | ,181 | 2,404 | 1 | ,121 | 1,324 | ,929 | 1,887 |  |
|  | ND_Suicide | ,102 | ,280 | ,132 | 1 | ,717 | 1,107 | ,640 | 1,916 |  |
|  | ND_Frustration | -,013 | ,178 | ,006 | 1 | ,940 | ,987 | ,696 | 1,398 |  |
|  | ND_Joy | ,188 | ,163 | 1,327 | 1 | ,249 | 1,207 | ,876 | 1,663 |  |
|  | ET_Anxiety | -,009 | ,056 | ,026 | 1 | ,871 | ,991 | ,888 | 1,106 |  |
|  | ET_Anger | ,375 | ,066 | 32,397 | 1 | ,000 | 1,454 | 1,278 | 1,655 |  |
|  | Age | ,000 | ,007 | ,001 | 1 | ,970 | 1,000 | ,986 | 1,015 |  |
|  | Gender | -,242 | ,247 | ,961 | 1 | ,327 | ,785 | ,484 | 1,273 |  |
|  | Epilepsy type | ,663 | ,254 | 6,844 | 1 | ,009 | 1,941 | 1,181 | 3,190 |  |
|  | Remission 12 month | ,145 | ,272 | ,281 | 1 | ,596 | 1,156 | ,677 | 1,971 |  |
|  | ASM number | -,083 | ,184 | ,205 | 1 | ,651 | ,920 | ,641 | 1,321 |  |
|  | Constant | -3,995 | ,823 | 23,553 | 1 | ,000 | ,018 |  |  |  |
|  | | | | | | | | | | |

# Regression model for LAEP item „Restlessnes“

|  | | | | | | | | | |
| --- | --- | --- | --- | --- | --- | --- | --- | --- | --- |
|  | | Coefficient B | Standard error | Wald | df | Sig. | Exp(B) | 95% Confidence interval for EXP(B) | |
|  |  |  |  |  |  |  |  | Lower value | Upper value |
| Schritt 1^a^ | ND_Struggle | ,470 | ,130 | 13,143 | 1 | ,000 | 1,599 | 1,241 | 2,061 |
|  | ND_Wrong | ,082 | ,176 | ,217 | 1 | ,642 | 1,085 | ,768 | 1,533 |
|  | ND_Guilt | ,376 | ,190 | 3,903 | 1 | ,048 | 1,456 | 1,003 | 2,115 |
|  | ND_Suicide | -,027 | ,300 | ,008 | 1 | ,928 | ,973 | ,540 | 1,753 |
|  | ND_Frustration | ,307 | ,179 | 2,945 | 1 | ,086 | 1,359 | ,957 | 1,929 |
|  | ND_Joy | ,363 | ,171 | 4,505 | 1 | ,034 | 1,437 | 1,028 | 2,009 |
|  | ET_Anxiety | ,086 | ,057 | 2,304 | 1 | ,129 | 1,090 | ,975 | 1,218 |
|  | ET_Anger | -,003 | ,065 | ,003 | 1 | ,958 | ,997 | ,877 | 1,133 |
|  | Age | ,007 | ,007 | 1,007 | 1 | ,316 | 1,007 | ,993 | 1,021 |
|  | Gender | ,199 | ,228 | ,758 | 1 | ,384 | 1,220 | ,780 | 1,908 |
|  | Epilepsy type | -,205 | ,234 | ,768 | 1 | ,381 | ,814 | ,515 | 1,289 |
|  | Remission 12 month | -,272 | ,250 | 1,181 | 1 | ,277 | ,762 | ,467 | 1,244 |
|  | ASM number | ,131 | ,174 | ,569 | 1 | ,451 | 1,140 | ,811 | 1,602 |
|  | Constant | -2,918 | ,750 | 15,134 | 1 | ,000 | ,054 |  |  |
|  | | | | | | | | | |

# Regression model for LAEP item „Tiredness“

|  | | | | | | | | | |
| --- | --- | --- | --- | --- | --- | --- | --- | --- | --- |
|  | | Coefficient B | Standard error | Wald | df | Sig. | Exp(B) | 95% Confidence interval for EXP(B) | |
|  |  |  |  |  |  |  |  | Lower value | Upper value |
| Schritt 1^a^ | ND_Struggle | 1,234 | ,205 | 36,285 | 1 | ,000 | 3,436 | 2,299 | 5,133 |
|  | ND_Wrong | ,263 | ,257 | 1,048 | 1 | ,306 | 1,301 | ,786 | 2,151 |
|  | ND_Guilt | ,176 | ,259 | ,462 | 1 | ,497 | 1,192 | ,718 | 1,979 |
|  | ND_Suicide | -,376 | ,421 | ,799 | 1 | ,371 | ,686 | ,301 | 1,566 |
|  | ND_Frustration | ,329 | ,271 | 1,473 | 1 | ,225 | 1,390 | ,817 | 2,366 |
|  | ND_Joy | ,296 | ,255 | 1,348 | 1 | ,246 | 1,345 | ,816 | 2,218 |
|  | ET_Anxiety | -,089 | ,077 | 1,347 | 1 | ,246 | ,915 | ,786 | 1,063 |
|  | ET_Anger | ,034 | ,092 | ,139 | 1 | ,709 | 1,035 | ,864 | 1,240 |
|  | Age | -,014 | ,008 | 2,916 | 1 | ,088 | ,986 | ,971 | 1,002 |
|  | Gender | ,319 | ,272 | 1,379 | 1 | ,240 | 1,376 | ,808 | 2,343 |
|  | Epilepsy type | ,031 | ,270 | ,013 | 1 | ,909 | 1,031 | ,608 | 1,749 |
|  | Remission 12 month | -,537 | ,283 | 3,598 | 1 | ,058 | ,585 | ,336 | 1,018 |
|  | ASM number | ,288 | ,215 | 1,793 | 1 | ,181 | 1,334 | ,875 | 2,033 |
|  | Constant | -1,912 | ,895 | 4,563 | 1 | ,033 | ,148 |  |  |
|  | | | | | | | | | |

# Regression model for LAEP item „Unsteadiness“

|  | | | | | | | | | |
| --- | --- | --- | --- | --- | --- | --- | --- | --- | --- |
|  | | Coefficient B | Standard error | Wald | df | Sig. | Exp(B) | 95% Confidence interval for EXP(B) | |
|  |  |  |  |  |  |  |  | Lower value | Upper value |
| Schritt 1^a^ | ND_Struggle | ,989 | ,232 | 18,122 | 1 | ,000 | 2,690 | 1,706 | 4,241 |
|  | ND_Wrong | -,185 | ,285 | ,424 | 1 | ,515 | ,831 | ,475 | 1,452 |
|  | ND_Guilt | -,056 | ,306 | ,034 | 1 | ,855 | ,946 | ,519 | 1,722 |
|  | ND_Suicide | ,534 | ,396 | 1,819 | 1 | ,177 | 1,706 | ,785 | 3,710 |
|  | ND_Frustration | ,036 | ,279 | ,017 | 1 | ,896 | 1,037 | ,600 | 1,792 |
|  | ND_Joy | ,002 | ,258 | ,000 | 1 | ,994 | 1,002 | ,604 | 1,662 |
|  | ET_Anxiety | -,187 | ,099 | 3,572 | 1 | ,059 | ,829 | ,683 | 1,007 |
|  | ET_Anger | -,020 | ,103 | ,037 | 1 | ,847 | ,980 | ,802 | 1,199 |
|  | Age | ,038 | ,013 | 8,063 | 1 | ,005 | 1,038 | 1,012 | 1,066 |
|  | Gender | ,634 | ,420 | 2,277 | 1 | ,131 | 1,885 | ,827 | 4,293 |
|  | Epilepsy type | ,131 | ,510 | ,067 | 1 | ,796 | 1,141 | ,420 | 3,097 |
|  | Remission 12 month | -,971 | ,592 | 2,689 | 1 | ,101 | ,379 | ,119 | 1,209 |
|  | ASM number | ,483 | ,288 | 2,821 | 1 | ,093 | 1,621 | ,922 | 2,850 |
|  | Constant | -7,885 | 1,504 | 27,492 | 1 | ,000 | ,000 |  |  |
|  | | | | | | | | | |
